# Author Correction: Donor-acceptor bulk-heterojunction sensitizer for efficient solid-state infrared-to-visible photon up-conversion

**DOI:** 10.1038/s41467-024-53250-0

**Published:** 2024-10-23

**Authors:** Pengqing Bi, Tao Zhang, Yuanyuan Guo, Jianqiu Wang, Xian Wei Chua, Zhihao Chen, Wei Peng Goh, Changyun Jiang, Elbert E. M. Chia, Jianhui Hou, Le Yang

**Affiliations:** 1 Institute of Materials Research and Engineering (IMRE), Agency for Science, Technology and Research (A*STAR), 2 Fusionopolis Way, Singapore, 138634 Republic of Singapore; 2 State Key Laboratory of Polymer Physics and Chemistry, Institute of Chemistry Chinese Academy of Sciences, Beijing, 100190 P. R. China; 3 Division of Physics and Applied Physics, School of Physical and Mathematical Sciences, Nanyang Technological University (NTU), Singapore, 637371 Republic of Singapore; 4 Department of Materials Science & Engineering, National University of Singapore (NUS), 9 Engineering Drive 1, Singapore, 117575 Republic of Singapore

**Keywords:** Optoelectronic devices and components, Organic molecules in materials science, Materials for devices

Correction to: *Nature Communications* 10.1038/s41467-024-50177-4, published online 8 July 2024

The original version of this Article ommited some information from the Acknowledgements.

The correct version of the Acknowledgements is:

This research is supported by the Singapore National Research Foundation Fellowships (NRFF) (NRF-NRFF15-2023-0011) (L.Y.); the A*STAR Central Research Fund (L.Y.); the Young Individual Research Grant (YIRG) (OUNI230401dENTIRG) (L.Y.); A*STAR Graduate Academy studentships (X.W.C., L.Y.); and the Singapore Ministry of Education Academic Research Fund Tier 3 (MOE2018-T3-1-002) (Y.G., E.E.M.C.).

This has been corrected in both the PDF and HTML versions of the Article.

