# Supplementary material for: Donor-acceptor bulk-heterojunction sensitizer for efficient solid-state infrared-to-visible photon up-conversion
Source: Nat Commun. 2024 Jul 8;15:5719. doi: 10.1038/s41467-024-50177-4 (PMC11231359; doi:10.1038/s41467-024-50177-4)
Supplement: Supplementary file 1 — Supplementary Information [file 41467_2024_50177_MOESM1_ESM.pdf]

Donor-acceptor bulk-heterojunction sensitizer for efficient solid-state infrared-to-visible photon up-conversion

Pengqing Bi[1], Tao Zhang[2], Yuanyuan Guo[3], Jianqiu Wang[2], Xian Wei Chua[1], Zhihao Chen[2], Wei Peng Goh[1], Changyun Jiang[1], Elbert E. M. Chia[3], Jianhui Hou[2], Le Yang[1,4,*]

[1]Institute of Materials Research and Engineering (IMRE), Agency for Science, Technology and Research (A*STAR), 2 Fusionopolis Way, 138634, Singapore.
[2]State Key Laboratory of Polymer Physics and Chemistry, Institute of Chemistry Chinese Academy of Sciences, Beijing 100190, P. R. China.
[3]Division of Physics and Applied Physics, School of Physical and Mathematical Sciences, Nanyang Technological University (NTU), 637371, Singapore.
[4]Department of Materials Science & Engineering, National University of Singapore (NUS), 9 Engineering Drive 1, Singapore, 117575, Republic of Singapore.
*Corresponding author email: yang_le@imre.a-star.edu.sg

Supplementary Table of Contents

1. Supplementary Figs. 1-19.

2. Supplementary Tables 1 and 2.

Supplementary Figures

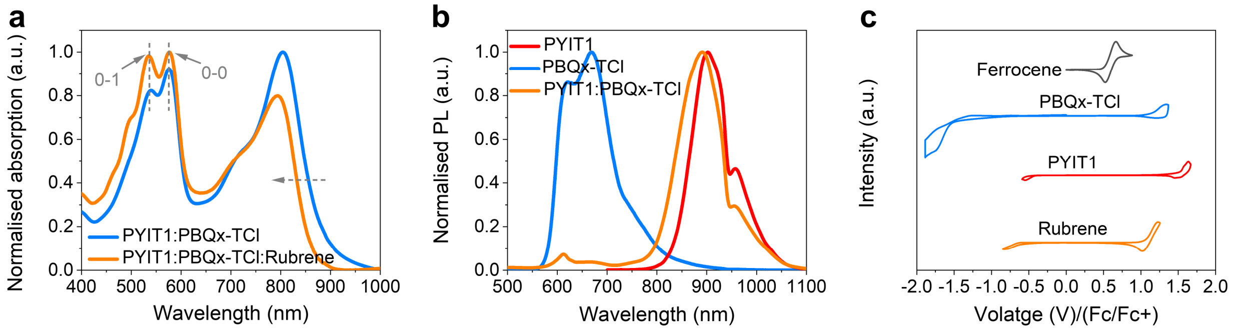

Supplementary Fig. 1 Materials basic properties. a The normalised absorption spectra of PYIT1:PBQx-TCl and PYIT1:PBQx-TCl:rubrene blend films. **b** The PL spectra of neat PYIT1, neat PBQx-TCl and PYIT1:PBQx-TCl blend films. **c** The cyclic voltammetry (CV) curves of neat PBQx-TCl, PYIT1 and rubrene films.

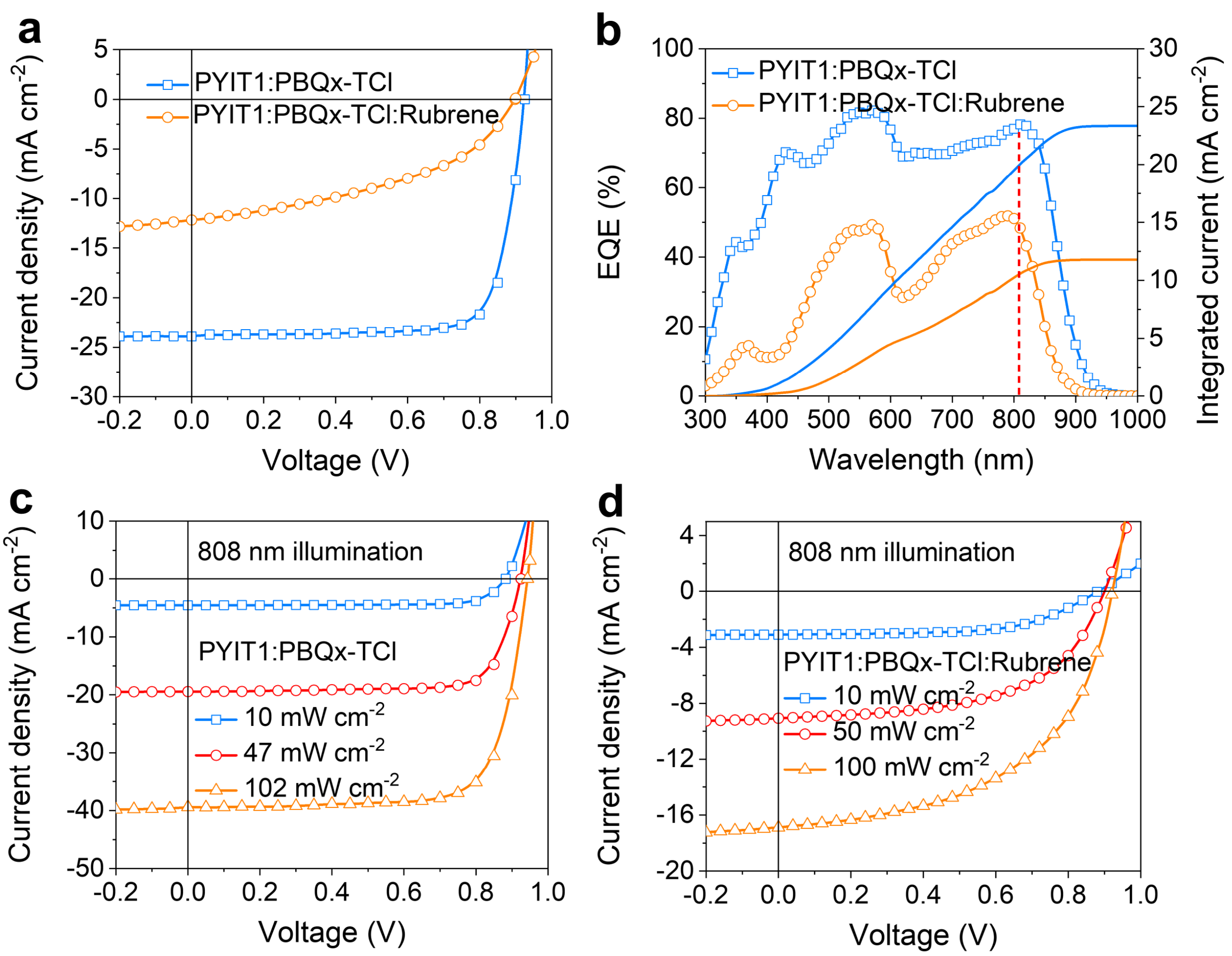

Supplementary Fig. 2 The performance of organic photovoltaic (OPV) cells. The (**a**) current-density-voltage (*J-V*) curves and (**b**) external quantum efficiency (EQE) spectra of the PYIT1:PBQx-TCl- and PYIT1:PBQx-TCl:rubrene-based OPV cells. The *J-V* curves of the (**c**) PYIT1:PBQx-TCl- and (**d**) PYIT1:PBQx-TCl:rubrene-based cells under 808 nm illuminations.

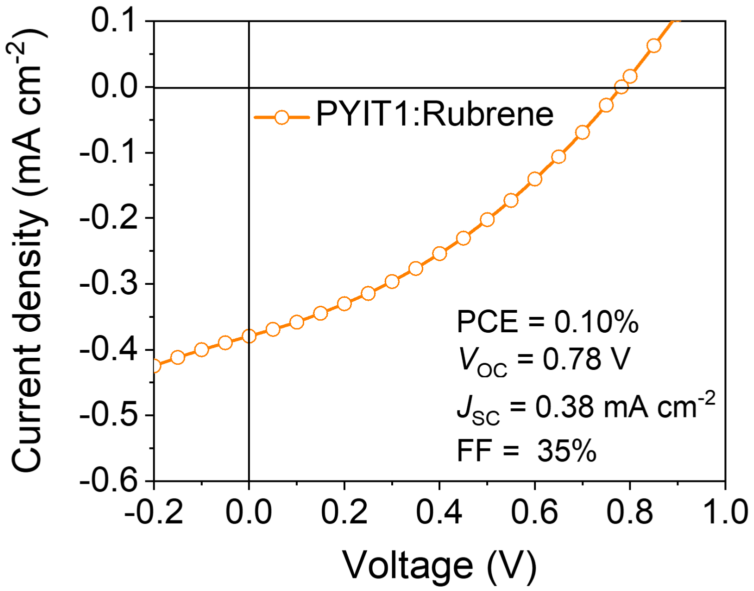

Supplementary Fig. 3 The performance of OPV cell. The *J*-*V* curve of the PYIT1:rubrene-based OPV cell under AM 1.5G (100 mW cm^{-2}).

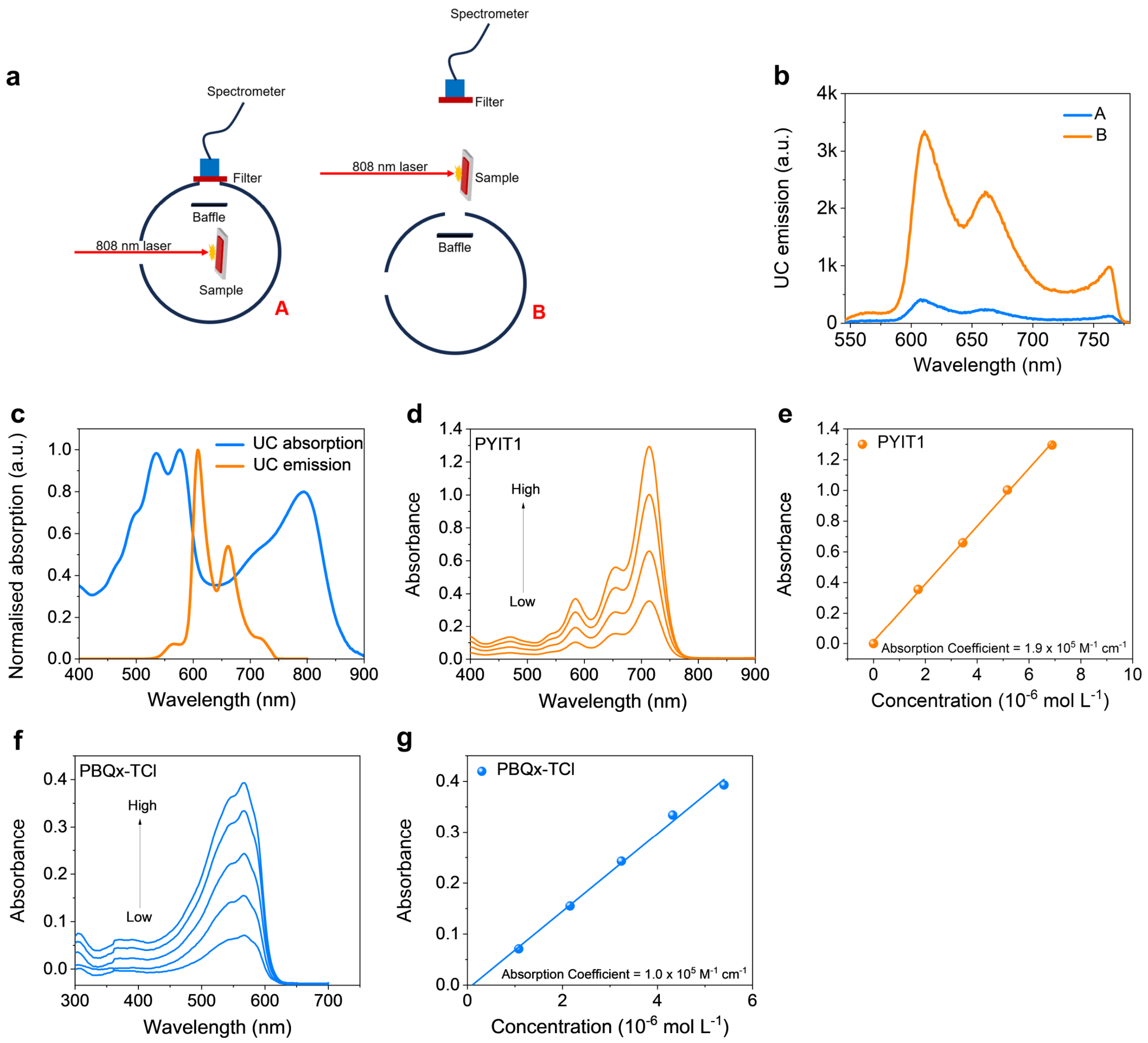

Supplementary Fig. 4 Reabsorption effect of the Triplet–triplet annihilation up-conversion (TTA-UC) device. a-b Experimental configurations for collecting UC spectra A (Sample in integrating sphere) and B (Sample out of integrating sphere). **c** The normalised absorption and UC emission spectra of the PYIT1:PBQx-TCl:rubrene:DBP film. **d-g** Absorption spectra of PYIT1 and PBQx-TCl with different concentrations in solutions and the fitted absorption coefficients.

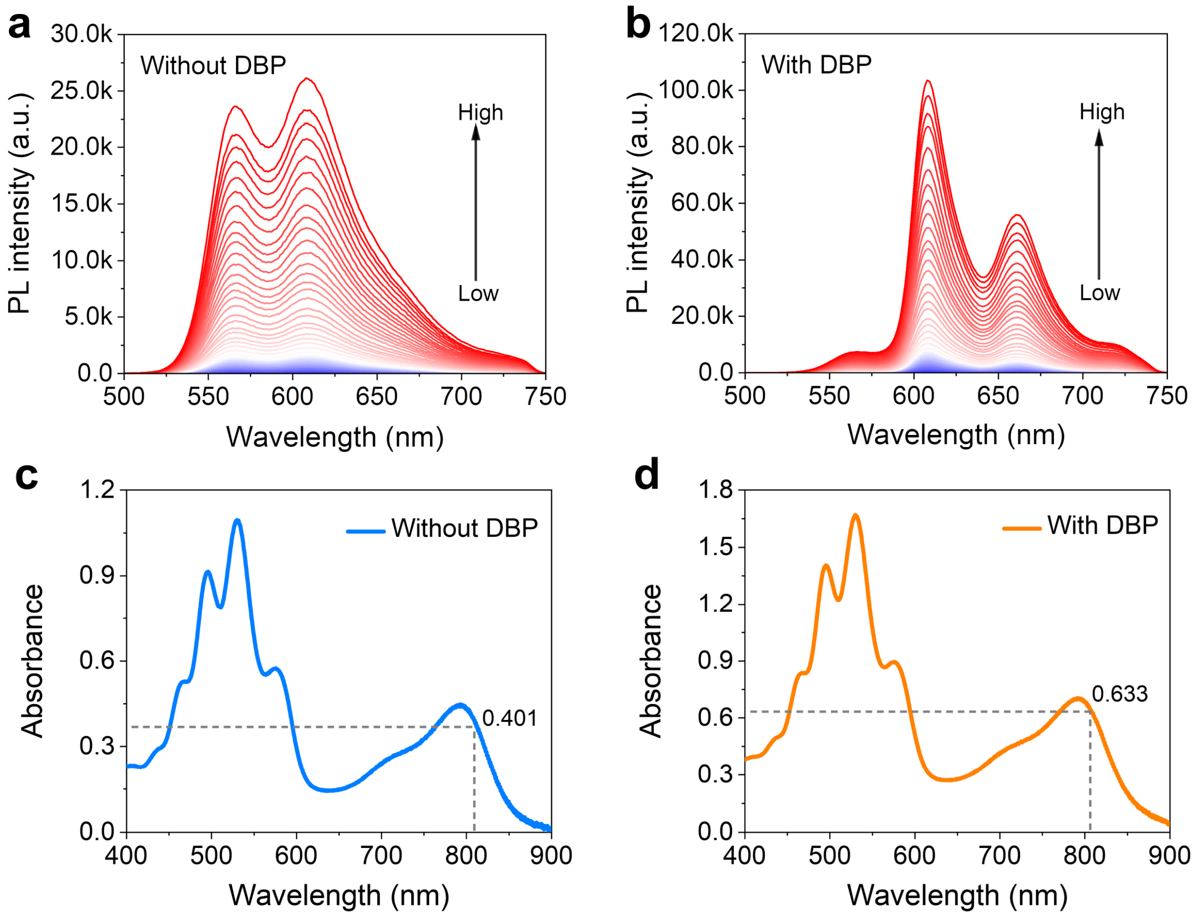

Supplementary Fig. 5 TTA-UC emission and absorption spectra. The PL spectra of (**a**) PYIT1:PBQx-TCl:rubrene- and (**b**) PYIT1:PBQx-TCl:rubrene:DBP-based TTA-UC devices with various excitation intensities. The absorption spectra of (**c**) PYIT1:PBQx-TCl:rubrene- and (**d**) PYIT1:PBQx-TCl:rubrene:DBP-based TTA-UC devices.

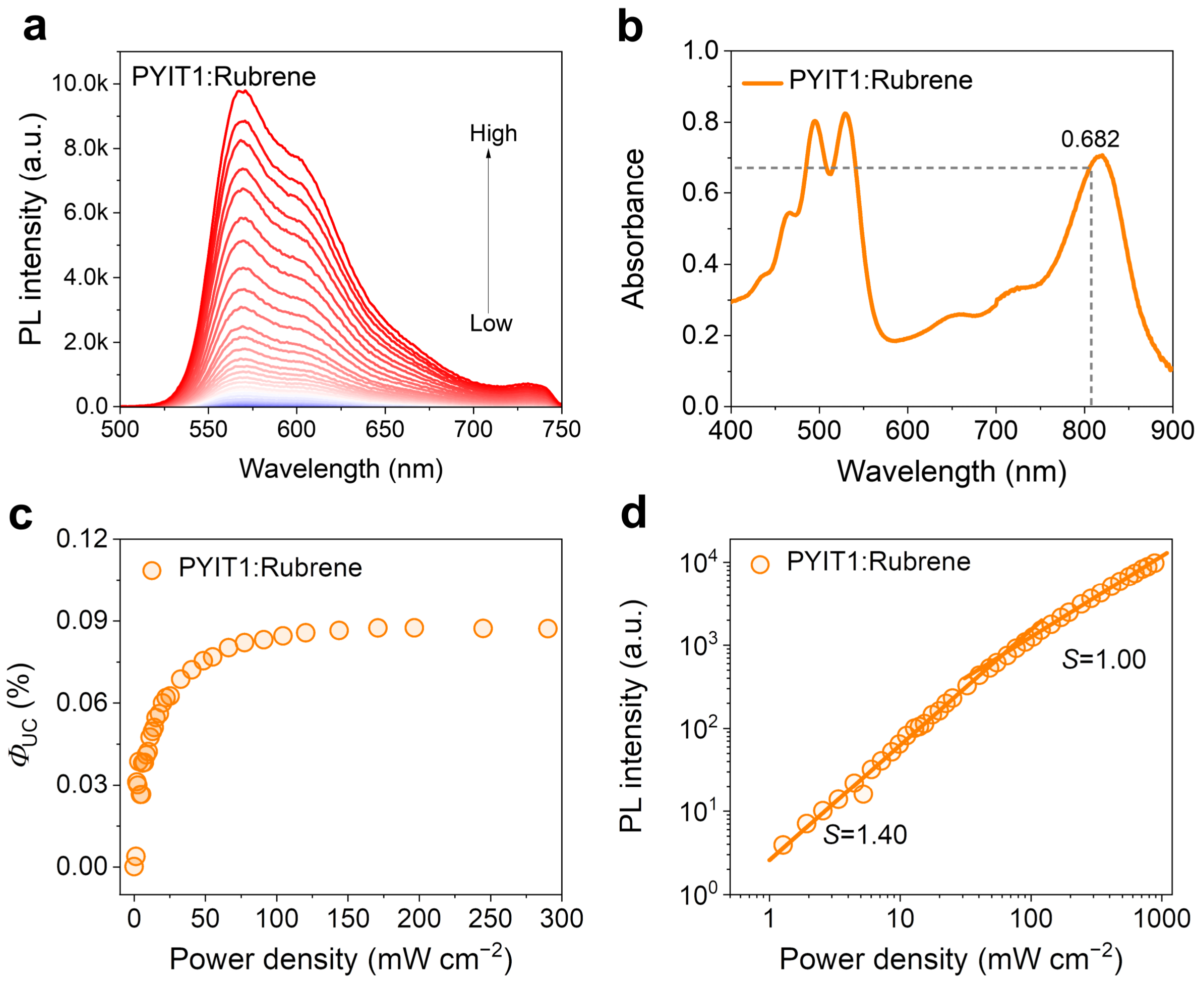

Supplementary Fig. 6 TTA-UC device performance. a The PL spectra of PYIT1:rubrene-based TTA-UC device with various excitation intensities. **b** The absorption spectra of PYIT1:rubrene-based TTA-UC device. **c** The Φ_{UC} of the PYIT1:rubrene-based UC device as a function of excitation power density. **d** The UC emission intensity of the PYIT1:rubrene-based UC device as a function of excitation power density.

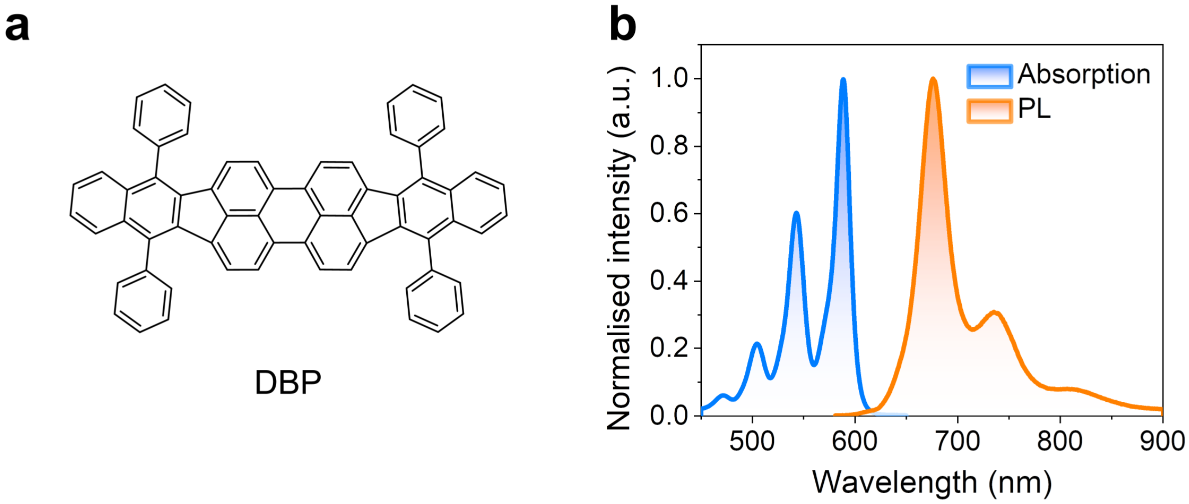

Supplementary Fig. 7 DBP properties. a The molecular structure of DBP. **b** The normalised absorption and PL spectra of DBP neat film.

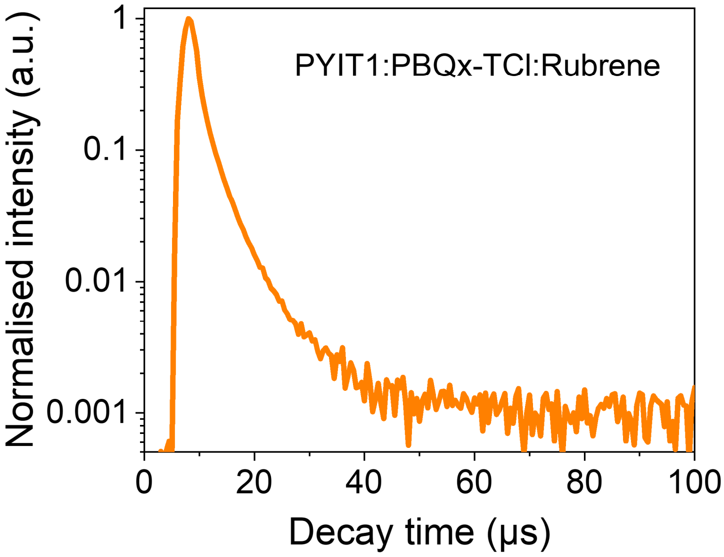

Supplementary Fig. 8 UC emission lifetime. TRPL decay curve of PYIT1:PBQx-TCl:rubrene film with excitation of 800 nm.

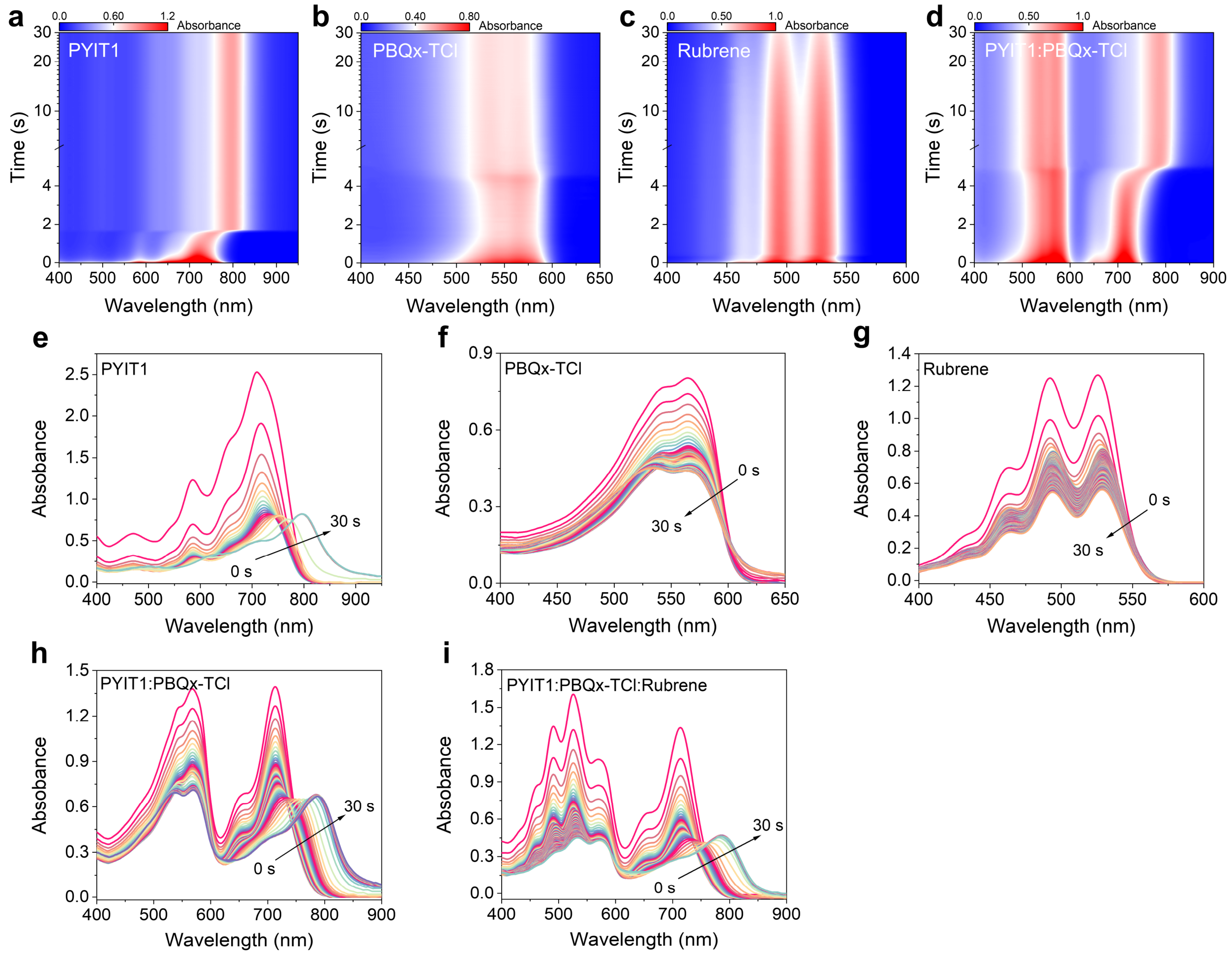

Supplementary Fig. 9 In-situ absorption. a-d Time-dependent contour maps of absorption spectra for the PYIT1, PBQx-TCl, rubrene and PYIT1:PBQx-TCl systems. **e-i** The absorption spectra of the PYIT1, PBQx-TCl, rubrene, PYIT1:PBQx-TCl, and PYIT1:PBQx-TCl:rubrene systems at various time points.

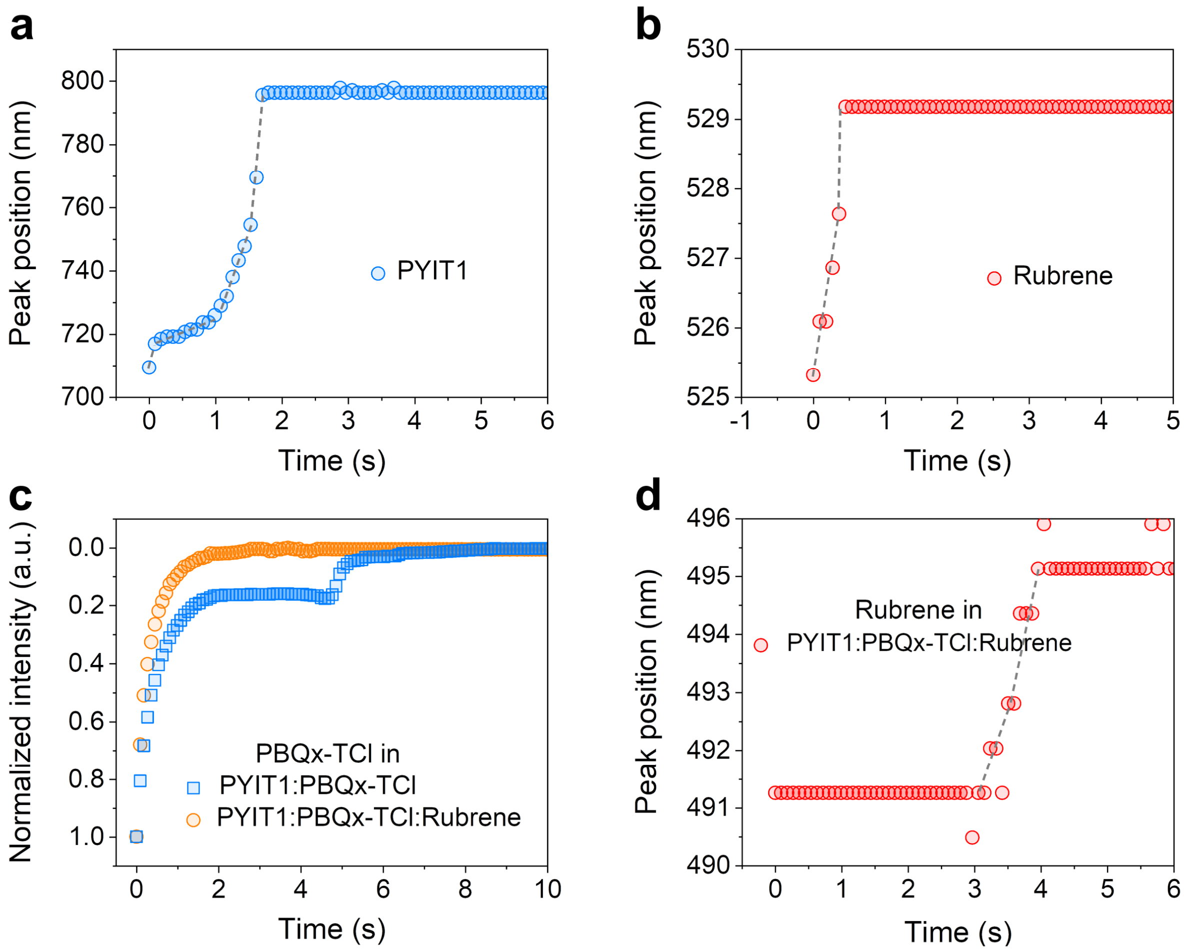

Supplementary Fig. 10 Phase transition processes. a Time evolution of the PYIT1 (0-0) absorption peak locations in neat PYIT system. **b** Time evolution of the rubrene absorption peak locations in neat rubrene system. **c** Time evolution of the absorption peak intensities of PBQx-TCl in PYIT1:PBQx-TCl and PYIT1:PBQx-TCl:rubrene systems. **d** Time evolution of the rubrene absorption peak locations in PYIT1:PBQx-TCl:rubrene system.

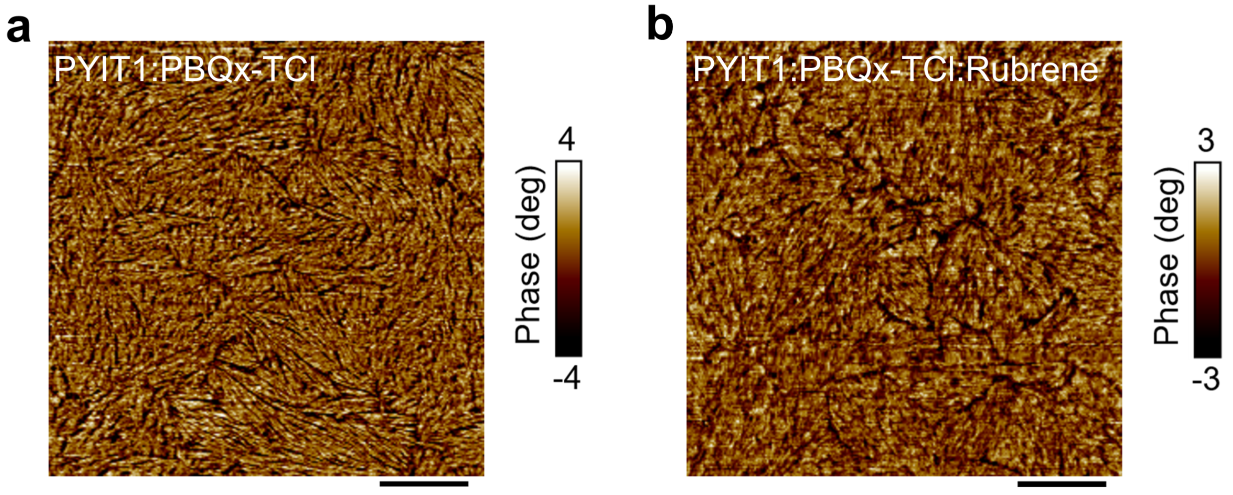

Supplementary Fig. 11 Atomic force microscope (AFM) phase images. AFM phase images of the (**a**) PYIT1:PBQx-TCl and (**b**) PYIT1:PBQx-TCl:rubrene films. The scale bar is 400 nm.

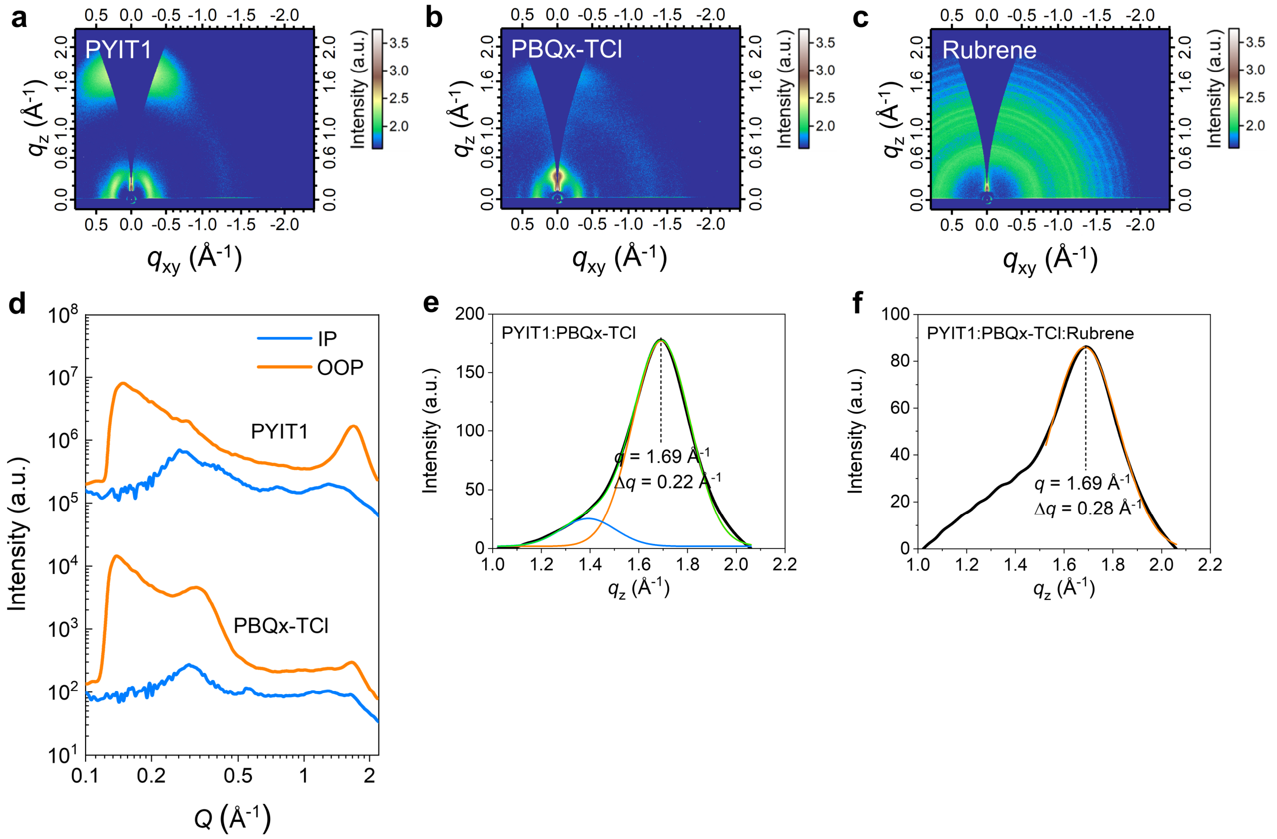

Supplementary Fig. 12 Crystalline properties of thin films. a-c 2D grazing incidence wide angle x-ray scattering (GIWAXS) patterns of the PYIT1, PBQx-TCl and rubrene films. The colour scales represent the log of diffraction intensity, in the unit of counts. **d** The 1D GIWAXS integration curves along IP and OOP directions. **e-f** Fitting curves of crystallisation peaks in PYIT1:PBQx-TCl and PYIT1:PBQx-TCl:rubrene films.

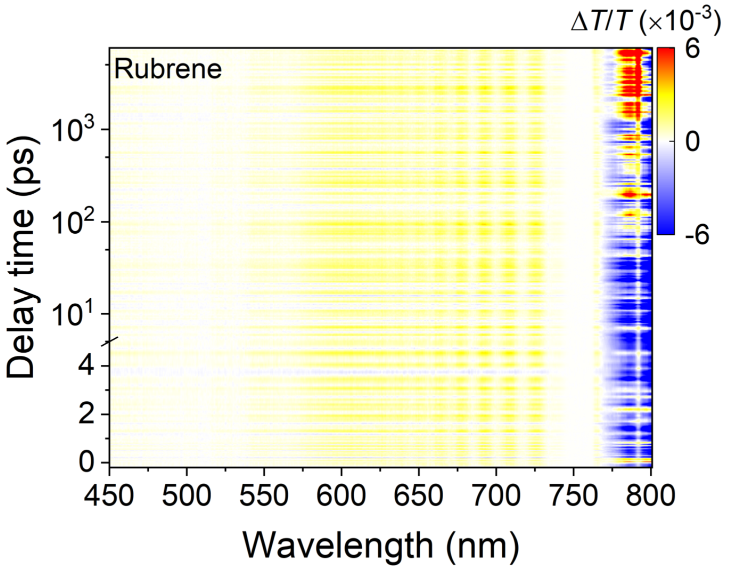

Supplementary Fig. 13 Transient absorption (TA) spectroscopic study. The TA image of rubrene neat film under 800 nm excitation.

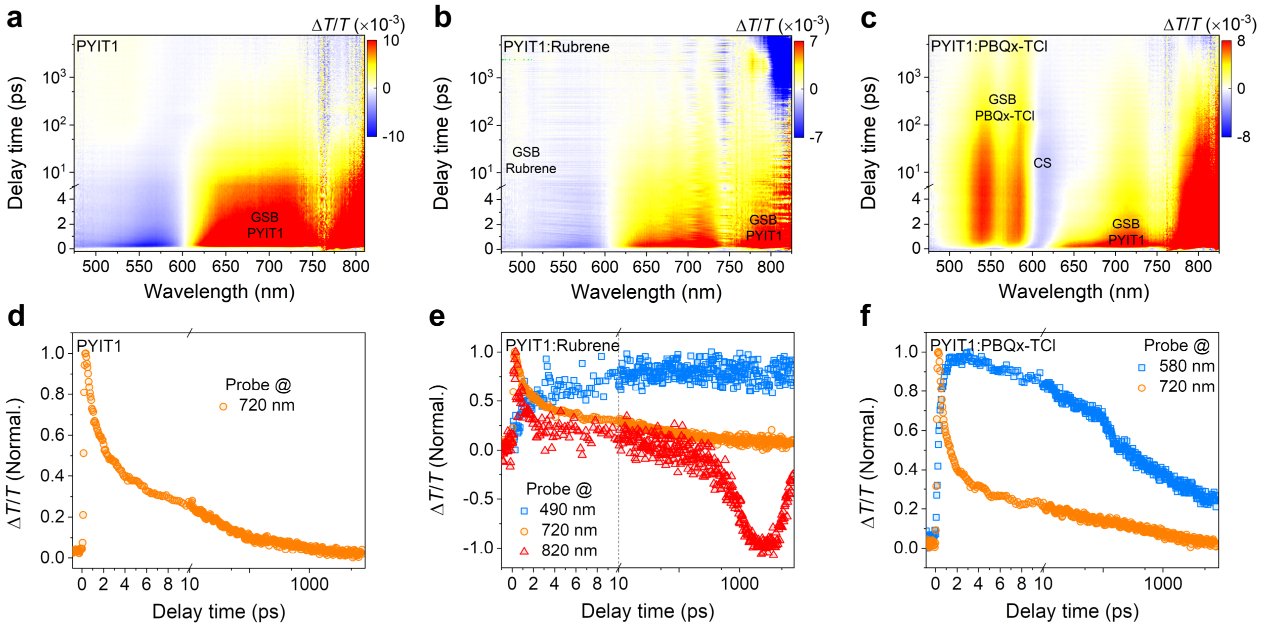

Supplementary Fig. 14 TA spectroscopic studies. The TA images of (**a**) PYIT1, (**b**) PYIT1:rubrene and (**c**) PYIT1:PBQx-TCl films. The normalised decay curves probed at various wavelengths recorded from (**d**) PYIT1, (**e**) PYIT1:rubrene and (**f**) PYIT1:PBQx-TCl films.

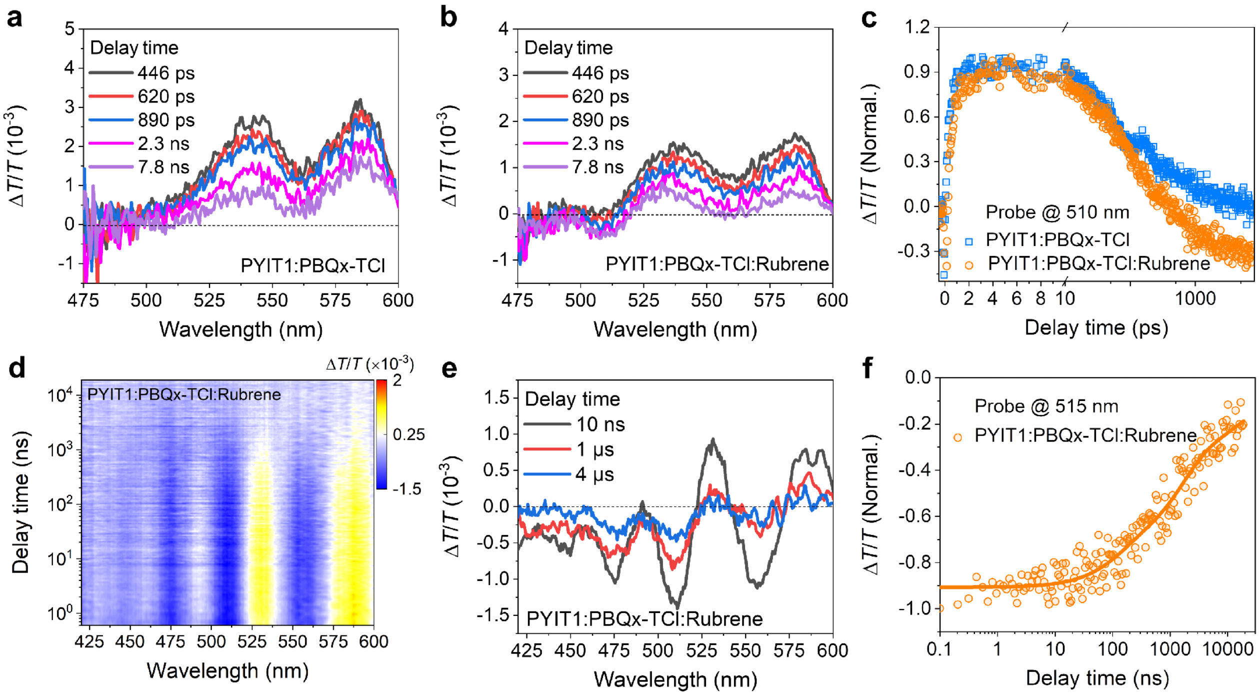

Supplementary Fig. 15 Triplet exciton of rubrene. TA spectra of (**a**) PYIT1:PBQx-TCl and (**b**) PYIT1:PBQx-TCl:rubrene films at different delay times. **c** The normalised decay curves probed at 510 nm recorded from PYIT1:PBQx-TCl and PYIT1:PBQx-TCl:rubrene films. **d** The ns-μs TA spectral map of PYIT1:PBQx-TCl:rubrene film. **e** The ns-μs TA spectra of PYIT1:PBQx-TCl:rubrene films at different delay times. **f** The normalised decay curves probed at 515 nm recorded from PYIT1:PBQx-TCl:rubrene films.

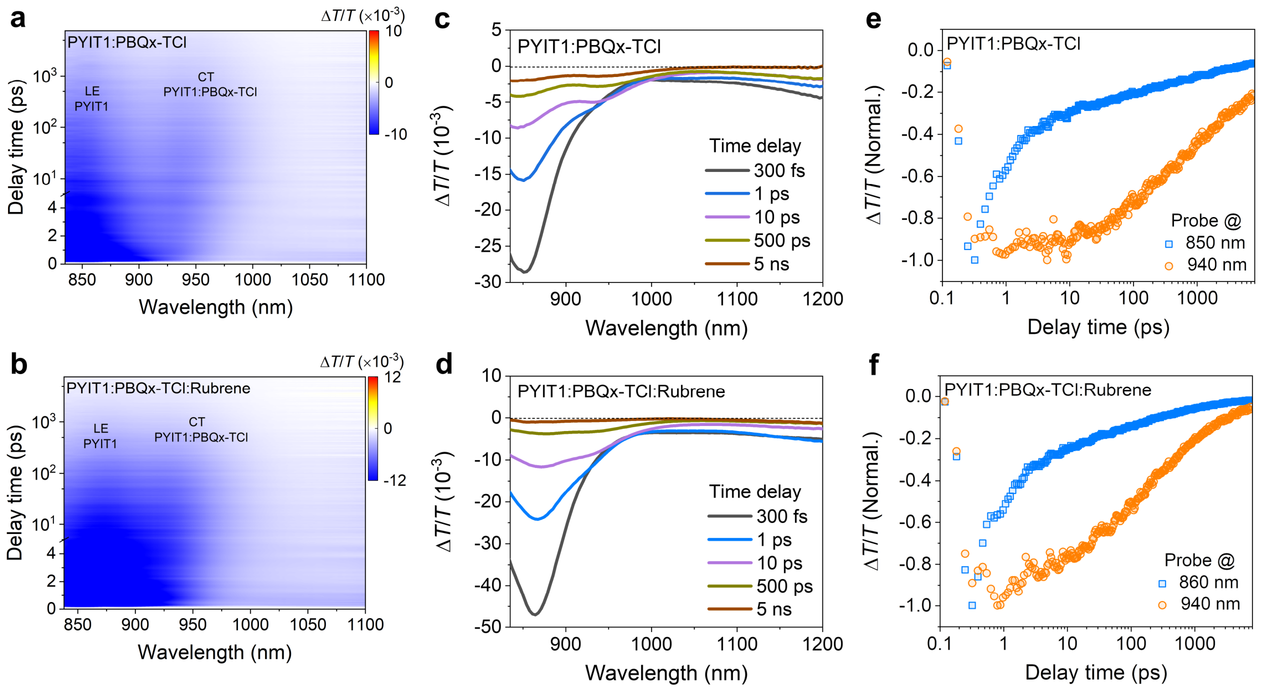

Supplementary Fig. 16 TA signals in NIR region. TA images of (**a**) PYIT1:PBQx-TCl and (**b**) PYIT1:PBQx-TCl:rubrene films. TA spectra of (**c**) PYIT1:PBQx-TCl and (**d**) PYIT1:PBQx-TCl:rubrene films at different delay times. The normalised decay curves probed at various wavelengths recorded from (**e**) PYIT1:PBQx-TCl and (**f**) PYIT1:PBQx-TCl:rubrene films.

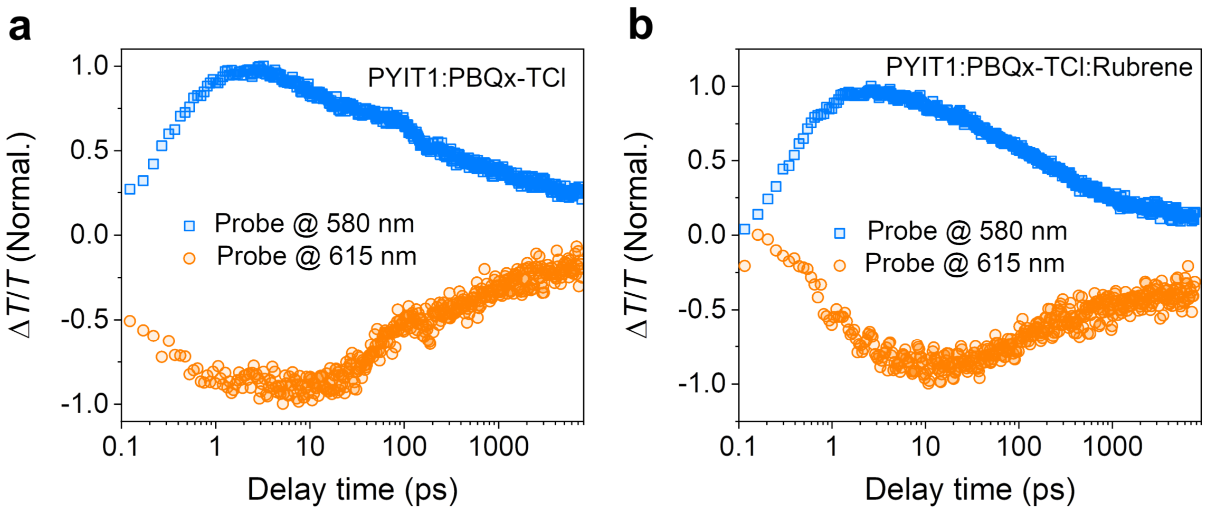

Supplementary Fig. 17 Charge separation (CS) states. The normalised decay curves probed at 580 nm and 615 nm recorded from (**a**) PYIT1:PBQx-TCl and (**b**) PYIT1:PBQx-TCl:rubrene films.

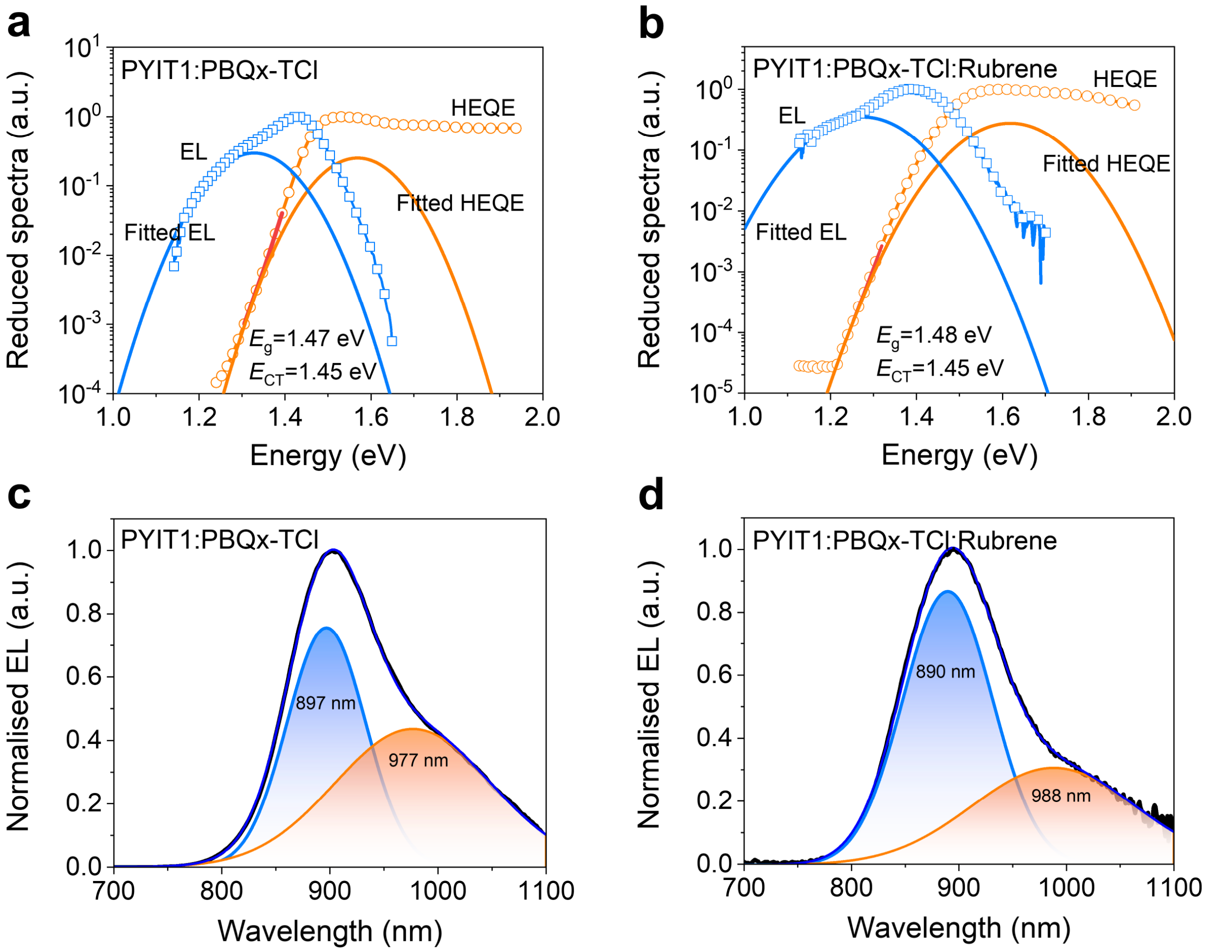

Supplementary Fig. 18 Charge transfer (CT) states of OPV cells. High-sensitivity EQE (HEQE) and electroluminescence (EL) spectra of the (**a**) PYIT1:PBQx-TCl- and (**b**) PYIT1:PBQx-TCl:rubrene-based OPV cells for determining the energy of charge transfer state (E_{CT}). EL spectra of the (**c**) PYIT1:PBQx-TCl- and (**d**) PYIT1:PBQx-TCl:rubrene-based OPV cells.

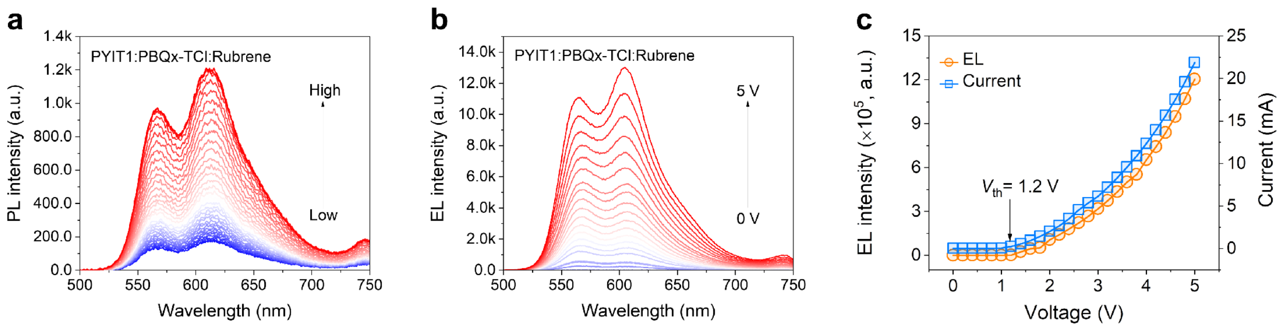

Supplementary Fig. 19 PL and EL emissions. a The UC emission spectra of PYIT1:PBQx-TCl:rubrene-based OPV cell at various excitation intensities. **b** The EL spectra of PYIT1:PBQx-TCl:rubrene-based OPV cell at various applied voltages. **c** The EL emission intensity and photocurrent of the PYIT1:PBQx-TCl:rubrene-based OPV cell at various applied voltages.

Supplementary Tables

Supplementary Table 1. Summary of photovoltaic parameters of the OPV cells under 1-sun illumination.

Active layers	V_{OC} (V)	J_{SC} (mA cm^{-2})	*Cal.* J_{SC} (mA cm^{-2})	FF (%)	PCE[a] (%)
PYIT1:PBQx-TCl	0.925	23.89	23.37	75.84	17.36 (17.03±0.21)
PYIT1:PBQx-TCl:Rubrene	0.902	12.09	11.81	47.75	5.21 (4.74±0.26)

[a] Average values with standard deviation were obtained from 6 devices.

Supplementary Table 2. Summary of photovoltaic parameters of the OPV cell under 808 nm illumination.

Devices	Power density (mW cm^{-2})	V_{OC} (V)	J_{SC} (mA cm^{-2})	FF (%)	PCE (%)
PYIT1:PBQx-TCl	10	0.883	4.56	78.75	31.70
	47	0.925	19.44	77.99	29.85
	102	0.943	39.41	75.63	27.56
PYIT1:PBQx-TCl:Rubrene	10	0.883	3.11	59.64	16.36
	50	0.901	9.08	56.02	9.17
	100	0.922	16.89	52.58	8.19
