# Supplementary material for: Donor-acceptor bulk-heterojunction sensitizer for efficient solid-state infrared-to-visible photon up-conversion
Source: Nat Commun. 2024 Jul 8;15:5719. doi: 10.1038/s41467-024-50177-4 (PMC11231359; doi:10.1038/s41467-024-50177-4)
Supplement: Supplementary file 3 — Solar Cells Reporting Summary [file 41467_2024_50177_MOESM3_ESM.pdf]

Corresponding author(s): Yang Le

Solar Cells Reporting Summary

Nature Portfolio wishes to improve the reproducibility of the work that we publish. This form is intended for publication with all accepted papers reporting the characterization of photovoltaic devices and provides structure for consistency and transparency in reporting. Some list items might not apply to an individual manuscript, but all fields must be completed for clarity.

For further information on Nature Research policies, including our data availability policy, see Authors & Referees.

Experimental design

Please check the following details are reported in the manuscript, and provide a brief description or explanation where applicable.

1. Dimensions

Area of the tested solar cells — [x] Yes [ ] No
Area of the tested solar cells is provided in methods.
Explain why this information is not reported/not relevant.

Method used to determine the device area — [x] Yes [ ] No
The method is provided in methods.
Explain why this information is not reported/not relevant.

2. Current-voltage characterization

Current density-voltage (J-V) plots in both forward and backward direction — [ ] Yes [x] No
Just J-V plot in forward direction since there is no hysteresis in organic solar cells.

Voltage scan conditions — [x] Yes [ ] No
The scan conditions are provided in methods.
Explain why this information is not reported/not relevant.

Test environment — [x] Yes [ ] No
Test environment is provided in methods.
Explain why this information is not reported/not relevant.

Protocol for preconditioning of the device before its characterization — [ ] Yes [x] No
No preconditioning protocol.
Explain why this information is not reported/not relevant.

Stability of the J-V characteristic — [ ] Yes [x] No
There is no corresponding testing equipment
Explain why this information is not reported/not relevant.

3. Hysteresis or any other unusual behaviour

Description of the unusual behaviour observed during the characterization — [ ] Yes [x] No
N.A.
Explain why this information is not reported/not relevant.

Related experimental data — [ ] Yes [x] No
N.A.
Explain why this information is not reported/not relevant.

4. Efficiency

External quantum efficiency (EQE) or incident photons to current efficiency (IPCE) — [x] Yes [ ] No
EQE spectra are measured and shown in the supporting information (SI).
Explain why this information is not reported/not relevant.

A comparison between the integrated response under the standard reference spectrum and the response measure under the simulator — [x] Yes [ ] No
The data are shown in the SI.
Explain why this information is not reported/not relevant.

April 2023

For tandem solar cells, the bias illumination and bias voltage used for each subcell

- [ ] Yes
- [x] No

Tandem OPV cell is not fabricated.

Explain why this information is not reported/not relevant.

5. Calibration

Light source and reference cell or sensor used for the characterization

- [x] Yes
- [ ] No

Relative information is provided in methods.

Explain why this information is not reported/not relevant.

Confirmation that the reference cell was calibrated and certified

- [x] Yes
- [ ] No

Relative information is provided in methods.

Explain why this information is not reported/not relevant.

Calculation of spectral mismatch between the reference cell and the devices under test

- [ ] Yes
- [x] No

We do not have the detailed information for the method.

Explain why this information is not reported/not relevant.

6. Mask/aperture

Size of the mask/aperture used during testing

- [x] Yes
- [ ] No

Size of the mask is provided in methods.

Explain why this information is not reported/not relevant.

Variation of the measured short-circuit current density with the mask/aperture area

- [x] Yes
- [ ] No

Data are shown in the SI.

Explain why this information is not reported/not relevant.

7. Performance certification

Identity of the independent certification laboratory that confirmed the photovoltaic performance

- [ ] Yes
- [x] No

The device performance is comparable to previous report, therefore no certification is required.

Explain why this information is not reported/not relevant.

A copy of any certificate(s)

- [ ] Yes
- [x] No

N.A.

Explain why this information is not reported/not relevant.

8. Statistics

Number of solar cells tested

- [x] Yes
- [ ] No

Number of cells tested is provided in the SI.

Explain why this information is not reported/not relevant.

Statistical analysis of the device performance

- [x] Yes
- [ ] No

Statistical results of the devices are shown in the SI.

Explain why this information is not reported/not relevant.

9. Long-term stability analysis

Type of analysis, bias conditions and environmental conditions

- [ ] Yes
- [x] No

This work does not investigate the stability issues.

Explain why this information is not reported/not relevant.
